# Lieutenant-Colonel John Tasman Waddell Leslie

**Published:** 1911-04-08

**Authors:** 


Lieutenant-Colonel John Tasman Waddell Leslie, C.I.E., Sanitary Commissioner with the Government of India, has died at Marseilles on his way home from India. He was educated at the University of Aberdeen, where he took the M.B. degree. He studied at Edinburgh, London, Munich, and Paris, and passed into the Indian Medical Service in 1884. In 1889 he was appointed secretary to the Inspector-General of Jails in Burma. He was junior Civil Surgeon of Rangoon during 1890, and two years later went to Calcutta as acting Chemical Examiner to Government and Professor of Chemistry at the Medical College. In 1893 he was appointed secretary to the Director-General of the Indian Medical Service and Sanitary Commissioner with the Government of India. In 1904 Colonel Leslie became the first wholetime Sanitary Commissioner. He organised the malarial conference opened by Lord Minto at Simla in October 1909, and was rewarded with the C.I.E.

